# Impact of the COVID-19 pandemic on skin cancer diagnosis: A population-based study.

**DOI:** 10.23889/ijpds.v7i3.1826

**Published:** 2022-08-25

**Authors:** Paul Nguyen, Yuka Asai, Timothy Hanna

**Affiliations:** 1 ICES Queen's; 2 Division of Dermatology, Department of Medicine, Queen’s University; 3 Division of Cancer Care and Epidemiology, Cancer Research Institute at Queen's University

## Objectives

The COVID-19 pandemic has been unprecedented and led to drastic reductions in nonurgent medical visits. Deferral of these visits may have critical health impact, including delayed diagnosis for melanoma and other skin cancers. We examined the influence of the pandemic on skin biopsy rates in a large population-based cohort.

## Approach

Using the universal health care claims dataset from Ontario, Canada, we examined skin biopsies from January 6, 2020 to September 27, 2020, and compared these to the same period for 2019. Those diagnosed with anogenital cancers, younger than 20 years, residing out-of-province and with lapses in coverage were excluded. The sensitivity and specificity of claims diagnoses were evaluated with a validated algorithm that identifies keratinocyte carcinoma (KC) in Ontario, and the Ontario Cancer Registry for melanoma cancer. Factors associated with biopsy during the early pandemic were investigated with modified Poisson regression.

## Results

A precipitous drop in total skin biopsies (down to 15% of expected), biopsies for KC (18%) and melanoma (27%) was seen with the onset of COVID-19 cases (p<0.01). Claims diagnoses were of high specificity for KC (99%), and for melanoma (98%), though sensitivity was less (61% and 28%, respectively). In adjusted analysis, the elderly (80+ years), females and residents of certain regions were less likely to be biopsied during the pandemic. Subsequently, there were substantial improvements in biopsy rates over 10 weeks. However, compared to 2019, a large backlog of expected cases still remained 28 weeks after lockdown (45,710 all biopsy, 9,104 KC and 595 melanoma).

## Conclusion

A drastic reduction in skin biopsies is noted early in the COVID-19 pandemic; this disproportionately affected the elderly, females and certain geographic regions. Though biopsies subsequently increased, a large backlog of cases remained after almost half a year. This will have implications for downstream care of skin cancer.

